# Book Review: Key Questions in Second Language Acquisition: An Introduction

**DOI:** 10.3389/fpsyg.2021.676360

**Published:** 2021-04-21

**Authors:** Mingjun Wu, Di Wu, Jingting Zeng

**Affiliations:** School of Foreign Languages, Jiangsu University of Science and Technology, Zhenjiang, China

**Keywords:** second language (L2) acquisition, input, instruction, explicit learning, individual differences, mental representation

Most learners of second language (L2), and even some teachers, those in the foreign language context in particular, hold the erroneous assumption that the data that learners adopt for the creation of the underlying “mental representation” of L2 are the rules they learn in textbooks or grammar guides and the drills or practices they are committed to. In their position, L2 acquisition amounts to rote learning of pedagogical textbook rules and new vocabulary. The key questions this volume raises is helpful to foster better understanding of successful L2 acquisition and to rectify the misconception.

This volume has eight chapters, which can fall into three parts. The first part (Chapter 1) raises the fundamental question of whether second language acquisition is similar to or different from first language acquisition. The second part (Chapters 2, 4, and 5) explores the process of second language acquisition from the initial state to the development until the ultimate attainment. The third part (Chapters 3, 6, 7, and 8) examines the different roles of input and output, instruction, explicit learning, and individual differences in L2 acquisition.

L2 acquisition originates from linguistics and L1 acquisition. The driving question is whether L1 and L2 acquisition is fundamentally the same or different. Although this question is raised in Chapter 1, due to the complex processes of L2 acquisition, it is not addressed until in the Epilog when the research has been reviewed on the major questions.

Chapter 2 is concerned with the issue of development and reviews various research of formal features of language, all of which are ordered and constrained by markedness, Universal Grammar, L1 knowledge, already acquired L2 knowledge, frequency, or general learning mechanism no matter whether they are inflectional morphemes, free morphemes, or a particular sentence structure, such as the acquisition of negation whose four developmental stages over time are: (1) simple negators *no/not* + a single word or a phrase to express negation (e.g., *no milk, not have coffee*); (2) moving a negator between a subject and verb (e.g., *I no want milk*); (3) correctly acquiring auxiliaries and modals with *not* but with the possibility of omitting copular *be* (*I don't have a book, I not crying*.); (4) using auxiliary verbs (*do, have*) and modals (e.g., *can, would, might*) with the correct negator *not* to produce nativelike sentences (e.g., *He doesn't like milk, I can't fix this car*).

Chapter 3 reviews research on the critical role that communicatively embedded input plays as the data for language learners to create an implicit linguistic system and on the three perspectives on the role of communicatively embedded output. L2 acquisition is actually the creation of a linguistic system in the mind/brain of the learner; therefore, it is a by-product of learners committed to the interpretation of language, that is, a by-product “of learners actively trying to comprehend language,” or “of input processing in which there is a focus on meaning.”

Chapter 4 explores research related to the initial state of L2 acquisition. There are three positions based on the generative tradition, Universal Grammar, the L1 and partial aspects of the L1, especially non-functional aspects. There are another three influential approaches to the initial state other than the Chomskyan tradition, namely usage-based models, Input Processing, and Processability Theory.

In Chapter 5, the question is addressed concerning L2 learners' steady-state mental representation. The Fundamental Difference Hypothesis is flawed in terms of both the process of L2 acquisition and the nature of the language system constrained and guided in the same way as L1. Furthermore, it is not clear whether the Critical Period Hypothesis can extend to L2 acquisition due to the difficulty in differentiating such factors as age, quantity and quality of input. It is most likely that the difference interfaces between syntax and other cognitive systems account for the apparent divergence from the native-speaker's knowledge and facility. Put it differently, “interfaces between different parts of language may cause long-term non-nativeness.”

Chapter 6 takes up the issue of the effect of formal instruction on L2 acquisition. Instruction plays little to no role in acquisition of L2 formal features since it does not alter the staged or ordered development. However, there has been controversy about how instruction affects the speed, short-term attainment, and ultimate attainment.

It is generally acknowledged that the outcome of L2 acquisition is to build an implicit linguistic system over time. Chapter 7 investigates the debate concerning explicit learning and explicit processes, and whether explicit learning impacts the development of implicit knowledge in L2 acquisition. Explicit learning seems to have limited effect on the acquisition of L2 formal features even in processing simple sentences.

Chapter 8 covers individual differences both in L1 acquisition and in L2 acquisition manifested as motivation, attitude, aptitude for learning, and working memory. Individual differences affect rate of L2 acquisition because it influences the access to input. However, individual differences do not change the cognitive processes in L2 acquisition.

Overall, this introductory book has several impressive and striking features in the organization, its language and content. The structure of this volume is unique in that it is not structured around topics or theories, but around eight key questions. Moreover, the structure of each chapter is unique in that it consists of recap, following-up and the three kinds of boxes: “Consider this…,” call-outs, and a special feature concerning social factors, in addition to main texts.

The language is quite reader-friendly without a “scholarly style and tone”; therefore, novice students get along with the volume with ease even though they have no background in any area related to L2 acquisition. The conclusion drawn after reviewing relevant research about key questions is convincing. For example, VanPatten et al. conclude that nativelikeness is not an all-or-nothing proposition. A learner could be nativelike in some aspects of the language but not nativelike in others, nativelike in some language use but not nativelike in other language use; some L2 learners become nativelike; most don't.

Despite the above strengths, this book is not without its shortcomings. The layout of the book appears to be more logical to unfold key questions in the order of origins of L2 acquisition, the initial state, development, ultimate attainment, the role of input and other factors, such as instruction, explicit learning and individual differences. What is more, some mistakes occur while the authors do their best to simplify the complex concepts. Firstly, complementizer is misinterpreted in that complementizers are words like *that, if* and *for* (Radford, 2004), but *which* and *who* are interrogative expressions (Radford, 2004). Secondly, wh-movement structure is incorrect in the diagram in that the land site of wh-expression *which book* is Spec of CP, but not the head C. Finally, the elaboration of Overt Pronoun Constraint is not accurate. Campos (1992) claims that an overt pronoun in a syntactic context where null pronouns are not allowed, as in the case of object of prepositions, can have a quantified expression (such as *everyone, someone, no one*) or a *wh-*phrase (e.g., *who, which*) as its antecedent:

Todo el mundo_i_ dice que el presidente habla de él_i_.

‘Everyone says that the president speaks of him.’

In a nutshell, the key issues in the field of L2 acquisition of this volume is well-articulated. This volume serves as an ideal introduction to such a varied audience as undergraduate and graduate students in SLA, language teachers and seasoned researchers by walking them through decades of research in SLA and providing them with insights and succinct overview. By virtue of the key questions and the compelling exemplary studies throughout the volume, the three leading scholars successfully guide readers, instill curiosity in them and spur their engagement with primary sources. Therefore, this volume is helpful to novice students, an administrator or anyone interested in L2 acquisition and can be adopted for classroom use.

## Author Contributions

All the three authors made discussions for the content, style, and evaluation of the book. MW drafted the part concerning origins, initial state, development and ultimate attainment of second language acquisition. DW drafted the part concerning input, instruction and explicit learning. JZ drafted the part concerning individual differences and revised the manuscript.

## Conflict of Interest

The authors declare that the research was conducted in the absence of any commercial or financial relationships that could be construed as a potential conflict of interest.
